# Anti-inflammatory activity of low molecular weight polysialic acid on human macrophages

**DOI:** 10.1038/srep16800

**Published:** 2015-11-19

**Authors:** Anahita Shahraz, Jens Kopatz, Rene Mathy, Joachim Kappler, Dominic Winter, Shoba Kapoor, Vlad Schütza, Thomas Scheper, Volkmar Gieselmann, Harald Neumann

**Affiliations:** 1 Institute of Reconstructive Neurobiology, University Hospital Bonn, University of Bonn, 53127 Bonn, Germany; 2 Institute of Biochemistry and Molecular Biology, University Hospital Bonn, University Bonn, Nussallee 11, 53115 Bonn, Germany; 3 Institute of Technical Chemistry, Leibnitz University Hannover, Callinstraße 5, 30167 Hannover, Germany

## Abstract

Oligosialic and polysialic acid (oligoSia and polySia) of the glycocalyx of neural and immune cells are linear chains, in which the sialic acid monomers are α2.8-glycosidically linked. Sialic acid-binding immunoglobulin-like lectin-11 (SIGLEC-11) is a primate-lineage specific receptor of human tissue macrophages and microglia that binds to α2.8-linked oligoSia. Here, we show that soluble low molecular weight polySia with an average degree of polymerization 20 (avDP20) interacts with SIGLEC-11 and acts anti-inflammatory on human THP1 macrophages involving the SIGLEC-11 receptor. Soluble polySia avDP20 inhibited the lipopolysaccharide (LPS)-induced gene transcription and protein expression of tumor necrosis factor-α (*Tumor Necrosis Factor Superfamily Member 2, TNFSF2*). In addition, polySia avDP20 neutralized the LPS-triggered increase in macrophage phagocytosis, but did not affect basal phagocytosis or endocytosis. Moreover, polySia avDP20 prevented the oxidative burst of human macrophages triggered by neural debris or fibrillary amyloid-β_1–42_. In a human macrophage-neuron co-culture system, polySia avDP20 also reduced loss of neurites triggered by fibrillary amyloid-β_1–42_. Thus, treatment with polySia avDP20 might be a new anti-inflammatory therapeutic strategy that also prevents the oxidative burst of macrophages.

*SIGLEC11* is a primate-lineage specific gene with a human-lineage specific expression profile that belongs to the human CD33-related SIGLEC receptor family^1^. Expression of SIGLEC-11 was detected in tissue macrophages such as Kupffer cells of the liver and microglia of the brain^1^,^2^. SIGLEC-11 recognizes α2.8-linked sialic acid (Sia), preferentially consisting of three monomers^1^,^2^, making it likely that oligosialic acid chains (oligoSia) represent ligands for SIGLEC-11. Artificial engagement of flag-tagged human SIGLEC-11 transduced into mouse microglia through crosslinking with flag-specific antibodies dampened the lipopolysaccharide (LPS)-induced pro-inflammatory gene transcription in mouse microglia^3^. Furthermore, the crosslinking of the flag-tagged SIGLEC-11 led to a decrease in phagocytosis of apoptotic cellular material^3^. In a co-culture system *SIGLEC11* gene transduced murine microglia showed protection of neurons against LPS-induced neurotoxicity^3^. Although the neuroprotective effect was abrogated when cultured neurons were treated with sialidases to deplete Sia from the glycocalyx^3^; the question whether oligoSia and/or possibly polysialic acid (polySia) can induce functional effects via SIGLEC-11 receptors remained unsolved.

PolySia in mammals is attached to glycoproteins of neural and immune cells such as neural cell adhesion molecule (NCAM; CD56), CD36 or neuropilin-2 (for review see^4^). The length of polymer chains measured as degree of polymerisation (DP) was shown to vary between DP10 to approximately DP200^4^,^5^. The mechanisms that regulate the addition of polySia to the carrier proteins as well as the mechanisms that control the chain length of added polymers are not fully understood. It has been demonstrated that polySia expression is tightly linked with the level of expressed polysialyltransferases^6^ and membrane associated neuraminidases^5^. Some neuroinvasive bacteria like *Escherichia coli* K1 can also produce a capsular polysaccharide, which in some substrains is chemically identical to polySia found in the human host^7^,^8^,^9^. By means of molecular mimicry the polySia coat supports escape of bacteria from immune recognition.

In this study, we analyzed the effect of soluble low molecular weight polySia on human SIGLEC-11 expressing THP1 cell derived macrophages. Low molecular weight polySia with an average DP20 exhibited an anti-inflammatory effect that was inhibited by a knock-down of the SIGLEC-11 receptor. Furthermore, low molecular weight polySia inhibited inflammatory phagocytosis without affecting basal phagocytosis and endocytosis. In addition, low molecular weight polySia completely prevented the phagocytosis associated oxidative burst of human macrophages. Moreover, in a human macrophage-neuron co-culture system, polySia avDP20 inhibited the radical mediated neurotoxicity triggered by fibrillary amyloid-β_1–42_.

## Results

### Low molecular weight polySia interacts with SIGLEC-11

**Figure 1:**
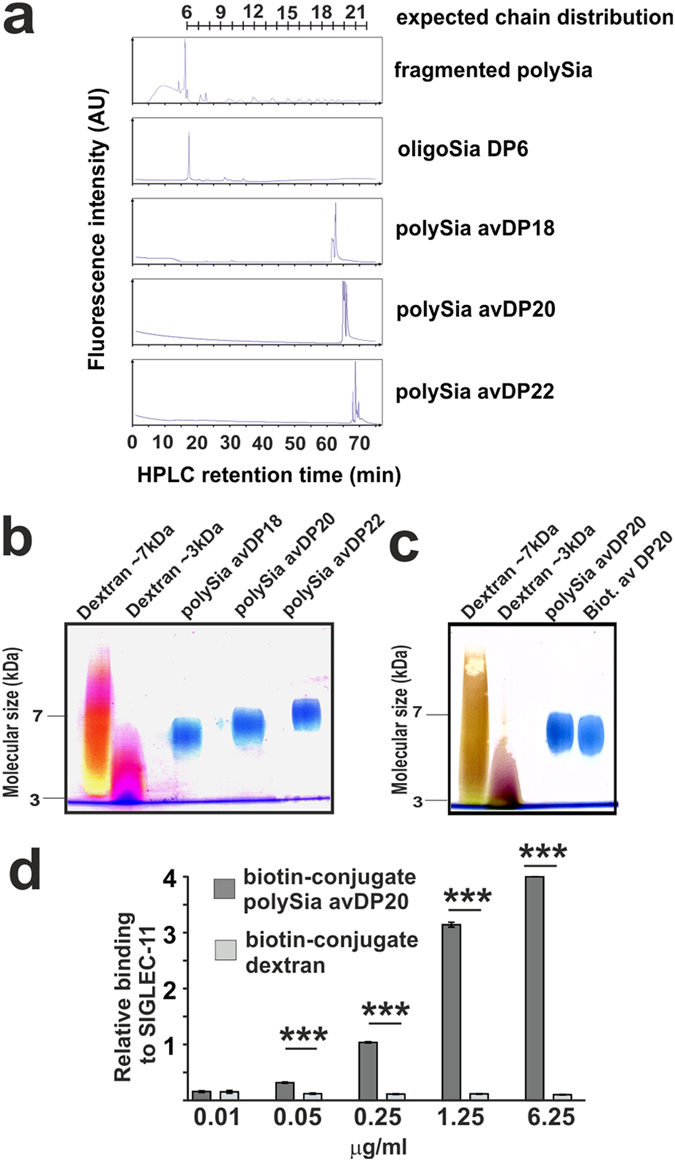
(**a**) HPLC analysis of oligoSia with a degree of polymerization of 6 (oligoSia DP6) and polySia with average degree of polymerization 18, 20 and 22 (polySia avDP18, avDP20 and avDP22) compared to hydrolyzed polySia. DMB-labeled oligoSia DP6, polySia avDP18, avDP20 and avDP22 were eluted by a NaCl buffer gradient. The HPLC retention times were monitored. (**b**) Polyacrylamide gel electrophoresis of polySia avDP18, avDP20 and polySia avDP22 visualized by stains-all solution. PolySia avDP20 showed a molecular weight of ~6 kDa. Dextran sulfate with molecular weights of ~3 kDa and ~7 kDa served as molecular weight controls. Representative image out of at least three independent experiments is shown. (**c**) Polyacrylamide gel electrophoresis of biotinylated polySia avDP20 (Biot. av DP20) visualized by stains-all solution. Majority of biotinylated polySia avDP20 is still intact after biotinylation. Representative image out of at least three independent experiments is shown. (**d**) ELISA to demonstrate the interaction between polySia with avDP20 and SIGLEC-11. SIGLEC-11 Fc-fusion protein was coated to an ELISA plate. Biotinylated polySia avDP20 (0.01, 0.05, 0.25, 1.25 and 6.25 μg/ml) or biotinylated dextran (0.01, 0.05, 0.25, 1.25 and 6.25 μg/ml) were added and detected by HRP conjugated streptavidin, followed by a TMB reaction. PolySia avDP20 bound to SIGLEC-11 Fc-fusion protein, while there was negligible binding of biotinylated dextran. Data are presented as relative binding (values of OD 450 measurements) mean +/− SEM of n = 3 independent experiments. ***p < 0.001; ANOVA followed by Bonferroni.

OligoSia in form of α2.8-linked Sia has been shown to bind to SIGLEC-11 receptors that are expressed on human tissue macrophages and microglia^1^,^2^, but the interaction of SIGLEC-11 with soluble polySia has not been analyzed. Therefore, we analyzed the binding of a soluble low molecular weight polySia to SIGLEC-11 by enzyme-linked immunosorbent assay (ELISA). To obtain low molecular weight polySia, a bacterial derived polySia (50–70 kDa) that was chemical identical to human polySia was fragmented by self-hydrolysis induced by mild heating^10^. Then, the obtained polySia fragments were separated by strong anion-exchange high-performance liquid chromatography (SAX-HPLC). A major sample representing a polySia fraction with a molecular weight between 4 and 8 kDa (here named avDP20) was isolated and selected as low molecular weight polySia for further functional experiments. The molecular size and purity of the fractioned polySia were confirmed by analytic high-performance liquid chromatography (HPLC) and polyacrylamide gel electrophoresis (Fig. 1a,b). The separation of polySia by SAX-HPLC allowed to separate different lengths of polySia (avDP18, avDP20 and avDP22), all representing compositions of polySia with defined range of molecular weights (Fig. 1a,b). To evaluate the binding between the selected polySia avDP20 and SIGLEC-11, a recombinant human SIGLEC-11 Fc-fusion (rhSIGLEC-11/Fc) protein was added to a protein-A coated plate. PolySia avDP20 was conjugated with biotin (Fig. 1c). Afterwards, different concentrations of biotinylated polySia avDP20 were added to the plate. Biotinylated dextran as a linear polysaccharide with a similar molecular weight (~5 kDa) was used as control. PolySia avDP20 bound to the rhSIGLEC-11/Fc fusion protein in a concentration dependent manner, while no binding of dextran was observed (Fig. 1d).

Thus, a direct binding of soluble polySia with avDP20 to SIGLEC-11 was detectable.

### Low molecular weight polySia acts anti-inflammatory on human macrophages via the SIGLEC-11 receptor

**Figure 2:**
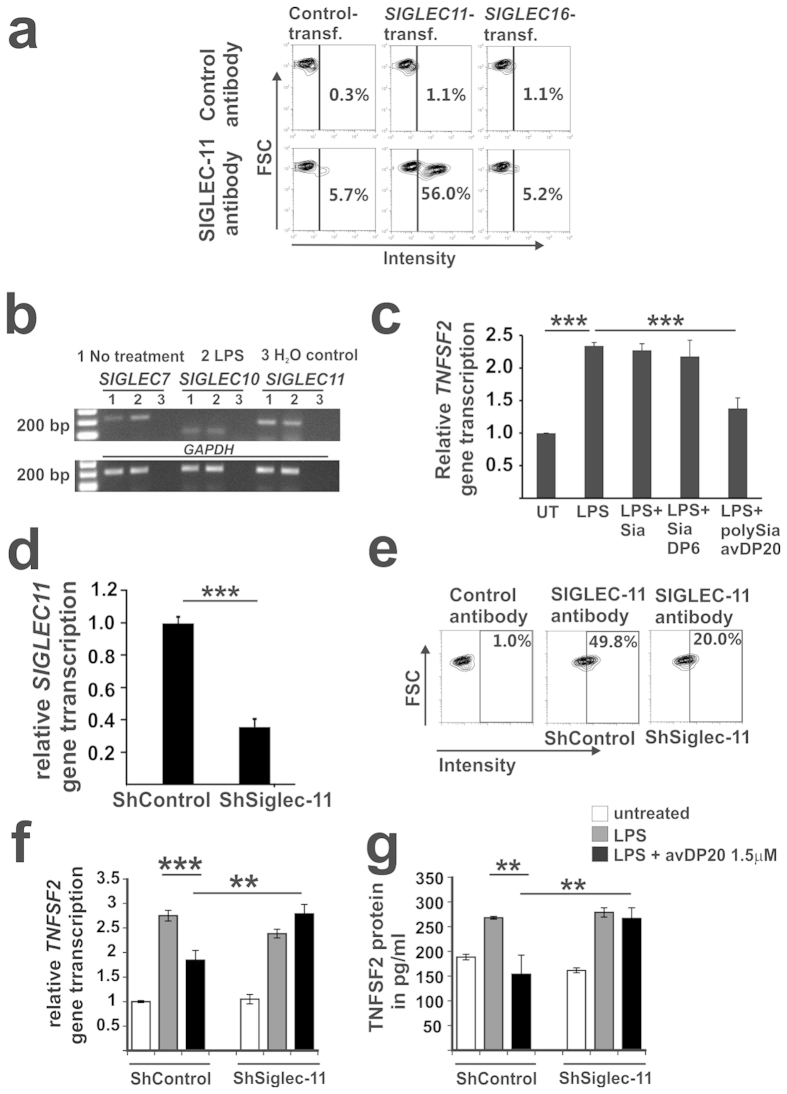
PolySia acts anti-inflammatory on human macrophages via SIGLEC-11 receptors. (**a**) Flow cytometry analysis of HEK-T-cells transfected with either control plasmid, *SIGLEC11* plasmid or *SIGLEC16* plasmid. GFP-positive cells were gated and stained with a SIGLEC-11 (clone 3 EH) antibody. SIGLEC-11 expression was detected on the *SIGLEC11* transfected cells. Control antibody: irrelevant isotype antibody. Representative histograms of n = 3 independent experiments. (**b**) Gene transcription of *SIGLEC7*, *SIGLEC10* and *SIGLEC11* in human macrophages. *SIGLEC11* shows the highest transcript levels. *GAPDH*: internal control. H_2_O: control without cDNA. Representative image of at least n = 3 independent experiments. (**c**) *TNFSF2* transcription of macrophages treated with LPS (1 μg/ml) alone or with LPS plus polySia avDP20 (1.5 μM), oligoSia with degree of polymerization 6 (SiaDP6; 1.5 μM) or sialic acid (Sia; 1.5 μM). Only polySia avDP20 reduced the LPS induced gene transcription of *TNFSF2*. Mean +/−  SEM of n = 3 independent experiments. ***p < 0.001; ANOVA followed by Bonferroni. (**d**) Gene transcripts for *SIGLEC11* were analyzed by qRT-PCR. Knock-down of *SIGLEC11* by short hairpin lentiviral vectors (ShSiglec-11) in macrophages reduced the gene transcription of *SIGLEC11*. ShControl: scrambled short hairpin vector. Mean +/− SEM of n = 4 independent experiments. ***p < 0.001; t-test. (**e**) SIGLEC-11 expression on macrophages was analyzed by flow cytometry. Knock-down of *SIGLEC11* by short hairpin vectors (ShSiglec-11) reduced the expression of SIGLEC-11. ShControl: scrambled short hairpin vector. (**f**) Effects of *SIGLEC11* knock-down on *TNFSF2* gene transcription. Macrophages were treated with LPS (1 μg/ml) alone or with LPS plus polySia avDP20 (1.5 μM). PolySia avDP20 reduced the *TNFSF2* gene transcription that were transduced with the control vector (ShControl), but failed to reduce the *TNFSF2* gene transcription levels after knock-down of *SIGLEC11* (ShSiglec-11). Mean +/− SEM of at least n = 3 independent experiments. **p < 0.01, ***p < 0.001; ANOVA followed by Bonferroni. (**g**) Effects of *SIGLEC11* knock-down on TNFSF2 protein secretion. Macrophages were treated 24 hours with LPS (1 μg/ml) alone or with LPS plus polySia avDP20 (1.5 μM). PolySia avDP20 reduced the TNFSF2 protein secretion that were transduced with the control vector (ShControl), but failed to reduce the TNFSF2 protein secretion levels after knock-down of *SIGLEC11* (ShSiglec-11). Mean +/− SEM of at least n = 3 independent experiments. **p < 0.01; ANOVA followed by Bonferroni.

Data from mouse microglia ectopically expressing the human *SIGLEC11* gene co-cultured with neurons suggested that sialic acids (Sias) of the neuronal glycocalyx might have anti-inflammatory activity^3^. To test whether polySia avDP20 act anti-inflammatory on macrophages, human macrophages differentiated from THP1 monocytes were used. The human macrophages showed protein expression of SIGLEC-11 as determined by flow cytometry with a newly generated monoclonal antibody directed against SIGLEC-11 (Fig. 2a). The specificity of the antibody was confirmed on HEK-T cells transfected with a human *SIGLEC11* overexpression plasmid. The SIGLEC-11 antibody (clone 3EH) bound to the *SIGLEC11* transfected cells; while *SIGLEC16* plasmid, as the closest homolog of *SIGLEC11*, and empty control plasmid transfected cells showed no substantial binding (Fig. 2a).

Next, we analyzed whether polySia avDP20 had anti-inflammatory effects on the gene transcription of the pro-inflammatory cytokine tumor necrosis factor-α (*Tumor Necrosis Factor Superfamily Member 2, TNFSF2*) that was induced in the macrophages by treatment with 1 μg/ml LPS. First, gene transcription of *SIGLEC7*, *SIGLEC10* and *SIGLEC11* was analyzed in human macrophages, obtained from THP1 monocytes, before and after treatment with LPS. Data show that human macrophages clearly transcribed *SIGLEC11*, while *SIGLEC10* was barely detected and *SIGLEC7* was inducible by LPS (Fig. 2b). Afterwards the effect of polySia avDP20 on gene transcription of *TNFSF2* was investigated. Results showed that polySia avDP20 prevented the LPS-induced gene transcription of *TNFSF2* at the concentration of 1.5 μM, while Sia as monomer or oligoSia with a DP of 6 had no effect (Fig. 2c). In detail, 1.5 μM polySia avDP20 antagonized the LPS-triggered *TNFSF2* gene transcription from 2.34 +/− 0.05 to 1.38 +/− 0.16 (Fig. 2c). Then, we asked whether the anti-inflammatory effect of polySia avDP20 on human macrophages was mediated via SIGLEC-11. In order to modify the expression of SIGLEC-11 on macrophages, we transduced cells with a lentiviral knock-down construct targeting *SIGLEC11*. Reduced gene transcription of *SIGLEC11* in the human macrophage was confirmed by qRT-PCR after lentiviral knock-down with a *SIGLEC11* targeting vector compared to a control vector (Fig. 2d). In detail, lentiviral knock-down of *SIGLEC11* reduced its relative gene transcript from 1 +/− 0.04 to 0.36 +/− 0.05 (Fig. 2d). Flow cytometry analysis also confirmed reduced cell surface expression of SIGLEC-11 on the human macrophages after lentiviral knock-down of *SIGLEC11* (Fig. 2e). Then, we analyzed whether the anti-inflammatory effect of polySia avDP20 on *TNFSF2* gene transcription was mediated via SIGLEC-11 receptors of human macrophages. Therefore, we stimulated the transduced macrophage cells (that received either *SIGLEC11* knock-down vector or control vector) with LPS (1 μg/ml) and treated them with 1.5 μM polySia avDP20 for 3 or 24 hours to assess transcription and protein level (Fig. 2f,g). Knock-down of *SIGLEC11* neutralized the anti-inflammatory effect of polySia avDP20 on the LPS-induced cytokine gene transcription of *TNFSF2* (Fig. 2f) and protein secretion of TNFSF2 (Fig. 2g). In detail, LPS-induced relative *TNFSF2* gene transcription was unchanged from 2.49 +/− 0.09 to 2.68 +/− 0.19 after lentiviral knock-down of *SIGLEC11*, while it was reduced from 2.75 +/− 0.11 to 1.85 +/− 0.2 after transduction with the control plasmid (Fig. 2f). Likewise, LPS-induced TNFSF2 protein secretion was unchanged from 278 +/− 9.3 to 266 +/− 21.2 pg/ml after lentiviral knock-down of *SIGLEC11*, while it was reduced from 268 +/− 2.8 to 153 +/− 38.9 after transduction with the control plasmid (Fig. 2g). Thus, polySia avDP20 acted anti-inflammatory on the LPS-induced *TNFSF2* gene transcription and protein secretion of human macrophages via the SIGLEC-11 receptor.

### Low molecular weight polySia acts anti-inflammatory on macrophages at nanomolar concentrations

**Figure 3:**
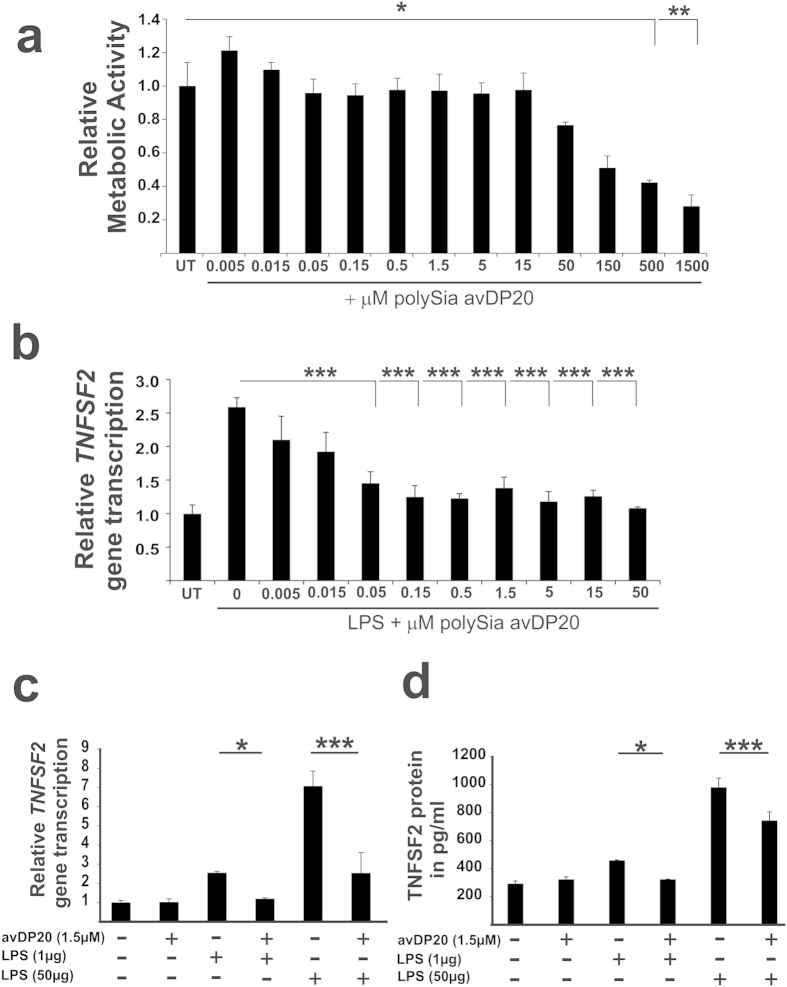
PolySia acts at nanomolar concentrations and prevents LPS-stimulated production of *TNFSF2*. (**a**) Metabolic activity of human macrophages was determined by the MTT-assay. Metabolic activity was reduced after addition of 500 μM and 1500 μM polySia avDP20 compared to the untreated control (UT), indicating reduced cell viability. Data are presented as mean +/− SEM of at least n = 3 independent experiments. *p < 0.05, **p < 0.01; ANOVA followed by Bonferroni. (**b**) Gene transcripts for *TNFSF2* of human macrophages after treatment with LPS (1 μg/ml) and different concentrations of polySia avDP20 for 3 hours. At a concentration of 0.05 μM or higher polySia avDP20 reduced the LPS-induced gene transcription of *TNFSF2*. Data are presented as mean +/− SEM of at least n = 3 independent experiments. ***p < 0.001; ANOVA followed by Bonferroni. (**c**) Gene transcription of *TNFSF2* after treatment with two different LPS concentrations (1 μg/ml and 50 μg/ml) and polySia avDP20 (1.5 μM) for 3 hours. PolySia avDP20 also reduced the gene transcription of *TNFSF2* induced by 50 μg/ml LPS. Data are presented as mean +/− SEM of at least n = 3 independent experiments. *p < 0.05, ***p < 0.001; ANOVA followed by Bonferroni. (**d**) TNFSF2 protein secretion after treatment with two different LPS concentrations (1 μg/ml and 50 μg/ml) and polySia avDP20 (1.5 μM) for 24 hours. PolySia avDP20 reduced the protein secretion of TNFSF2 induced by 50 μg/ml LPS. Data are presented as mean +/− SEM of at least n = 3 independent experiments. *p < 0.05, ***p < 0.001; ANOVA followed by Bonferroni.

Next, we tested a wider concentration range of polySia avDP20 on human macrophages. Therefore, we also analyzed the effect of polySia avDP20 on the metabolic activity of macrophages. Even relative high concentrations of polySia avDP20 up to 150 μM did not significantly interfere with cell viability as determined by the MTT-assay (Fig. 3a). The half maximum toxic concentration in the 24 hours MTT-assay was determined as TC50m = 148.78 μM. Next, we determined the half maximum effective concentration of polySia avDP20 leading to a 50% reduction of the *TNFSF2* gene transcripts induced in the human macrophages by 1 μg/ml LPS. This half effective concentration was determined as EC50m = 0.14 μM (Fig. 3b). Thus, the therapeutic index of polySia avDP20 on human macrophages, calculated as the ratio of the compound’s 50% toxic concentration to the compound’s 50% effective concentration, was TI(m/m) = TC50m/EC50m = 1062.71.

Next, we analyzed whether polySia avDP20 could also antagonize the *TNFSF2* gene transcription induced by higher concentration of LPS. Therefore, human macrophages were treated for 3 hours with 50 μg/ml LPS plus 1.5 μM polySia avDP20 (Fig. 3c). Treatment with 50 μg/ml LPS increased the gene transcription of *TNFSF2* from 1 +/− 0.08 to 7.3 +/− 0.8, while addition of 1.5 μM polySia avDP20 antagonized this increase to only 2.59 +/− 1.1 (p = 0.001; Fig. 3c).

Then, we asked whether polySia avDP20 also interferes with the LPS-stimulated TNFSF2 protein secretion of human macrophages. Macrophages were treated for 24 hours with LPS +/− 1.5 μM polySia avDP20; and then TNFSF2 protein secretion by macrophages was determined by ELISA (Fig. 3d). LPS (50 μg/ml) led to a production of 978.86 +/− 66.98 pg/ml TNFSF2 protein in the supernatant. However addition of polySia avDP20 (1.5 μM) reduced the secreted TNFSF2 protein to 741.06 +/− 62.01 pg/ml (p = 0.001; Fig. 3d).

Thus, polySia avDP20 showed a relative high therapeutic index on cultured human cells and acted anti-inflammatory on human macrophages at the lowest concentration of 0.05 μM without clear signs of toxicity up to 150 μM as determined by the MTT-assay.

### Low molecular weight polySia inhibits LPS-triggered phagocytosis of microbeads, but does not affect endocytosis of nanobeads

**Figure 4:**
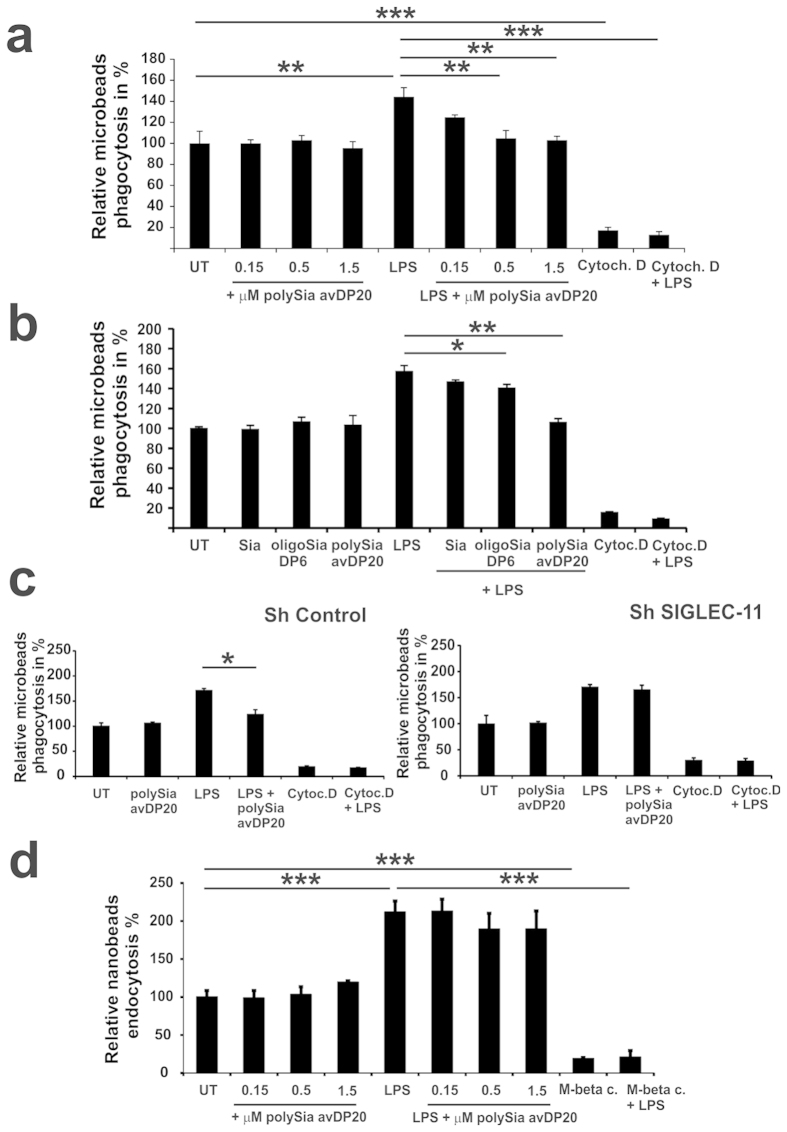
PolySia does not affect basal phagocytosis and homeostatic endocytosis. (**a**) Phagocytosis of microbeads as determined by flow cytometry. PolySia avDP20 did not change phagocytosis of microbeads. However, the LPS-induced increase in microbeads phagocytosis was antagonized by 0.5 μM and 1.5 μM polySia avDP20. The phagocytosis inhibitor cytochalasin D was used as control. Data are presented as mean +/− SEM of at least n = 3 independent experiments. **p < 0.01, ***p < 0.001; ANOVA followed by Bonferroni. (**b**) Phagocytosis of microbeads as determined by flow cytometry. Sia, oligoSia DP6 and polySia avDP20 did not change phagocytosis of microbeads. However, LPS-induced increase in microbead phagocytosis was antagonized by 1.5 μM polySia avDP20. The phagocytosis inhibitor cytochalasin D was used as control. Data are presented as mean +/− SEM of at least n = 3 independent experiments. *p < 0.05; **p < 0.01; ANOVA followed by Bonferroni. (**c**) Phagocytosis of microbeads by transduced THP1-derived macrophages as determined by flow cytometry. In control plasmid (Sh Control) transduced cells, polySia avDP20 was able to antagonize the effect of LPS on microbead phagocytosis. However, in SIGLEC11 knock-down plasmid (Sh SIGLEC-11) transduced macrophages, polySia avDP20 had no effect on LPS-induced microbead phagocytosis. The phagocytosis inhibitor cytochalasin D was used as control. Data are presented as mean +/− SEM of at least n = 3 independent experiments. * < 0.05; ANOVA followed by Bonferroni. (**d**) Endocytosis of nanobeads as determined by flow cytometry. PolySia avDP20 did not change the homeostatic basal nanobeads endocytosis. Furthermore, the inflammatory LPS-induced increase of nanobead endocytosis was not antagonized by polySia avDP20. The endocytosis inhibitor methyl-beta-cyclodextrin was used as control. Data are presented as mean +/− SEM of n = 3 independent experiments. ***p < 0.001; ANOVA followed by Bonferroni.

Since inhibitory Siglecs could increase endocytosis and reduce phagocytosis^11^, we analyzed the effect of polySia avDP20 on basal and inflammation triggered uptake of microbeads and nanobeads. Phagocytic uptake of microbeads was relatively increased from 100 +/− 11.4% to 144 +/− 8.8% after treatment with 1 μg/ml LPS (Fig. 4a). Treatment with 1.5 μM polySia avDP20 prevented this LPS-dependent increase in phagocytosis to 103 +/− 3.7% without interference with basal phagocytosis of macrophages (Fig. 4a). To compare the effect of different lengths of sialic acids, phagocytic uptake of microbeads was investigated after treatment of cells with 1.5 μM of Sia, oligoSia DP6 and polySia avDP20. Treatment with 1.5 μM polySia avDP20 reverted the LPS-triggered increase in phagocytosis from 157 +/− 2% to 106 +/− 4% without affecting basal macrophage phagocytosis (Fig. 4b). Then, we analyzed whether SIGLEC-11 is involved in the inhibitory effect of polySia avDP20 on the LPS-stimulated microbead phagocytosis. Lentiviral knock-down of *SIGLEC11* in the macrophages abolished the inhibitory effect of polySia avDP20 on phagocytosis compared to control plasmid transduced cells, which showed a reduction in microbeads phagocytosis from 171 +/− 4% to 124 +/− 9% (Fig. 4c). Next, we analyzed the effect of polySia avDP20 on endocytosis. Basal endocytosis of nanobeads was unchanged after treatment with polySia avDP20. Interestingly, also the LPS-triggered increase in phagocytosis of nanobeads from 100 +/− 8.1% to 212 +/− 14.3% was not significantly modulated by polySia avDP20 (Fig. 4d). Cytochalasin D and methyl-beta-cyclodextrin, which are inhibitors of phagocytosis and endocytosis, respectively, were used as controls (Fig. 4a–d).

Thus, polySia avDP20 does not affect basal phagocytosis and homeostatic endocytosis, but prevents the LPS-triggered increase in the inflammation associated phagocytosis.

### Low molecular weight polySia prevents the phagocytosis associated respiratory burst

**Figure 5:**
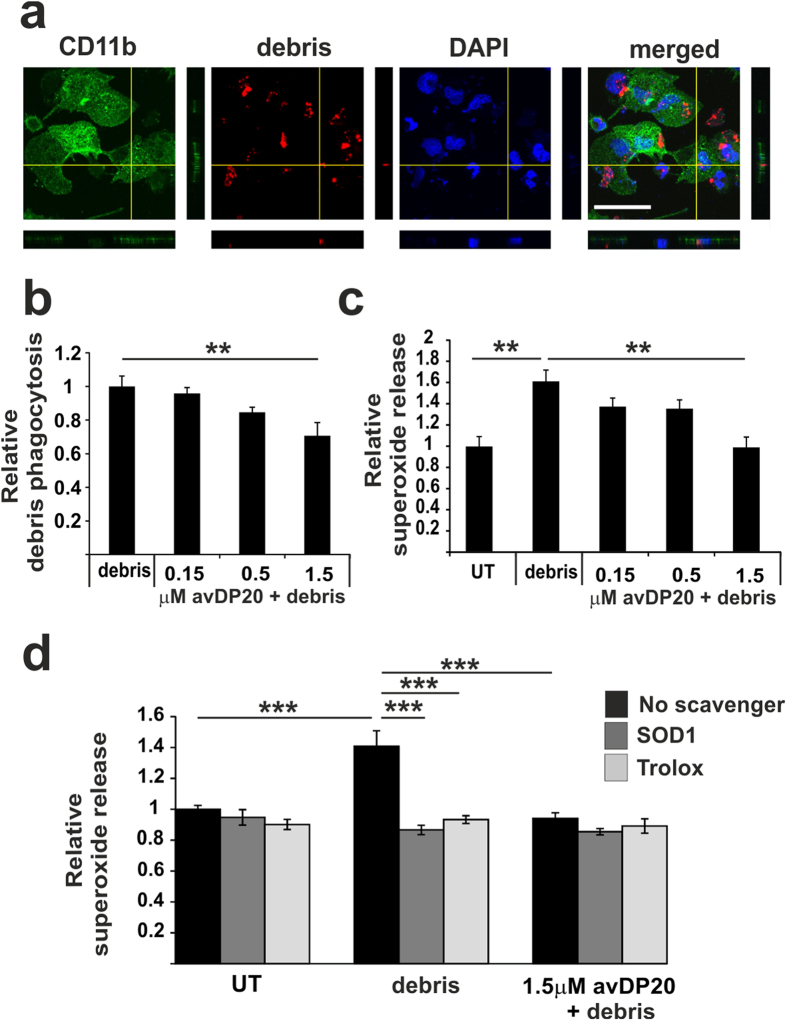
PolySia avDP20 reduced macrophage phagocytosis of neural debris and prevented the associated oxidative burst. (**a**) Phagocytosis of neural debris by human macrophages. Neural debris was labelled with a red fluorescent dye and added for 1.5 hour to macrophages. Cells were fixed and counter labelled with an antibody directed against CD11b. Confocal 3D-reconstruction of a macrophage cell that has ingested neural debris is shown. Nuclei are labelled by DAPI. Scale bar: 50 μm. (**b**) Quantification of the uptake of neural debris into macrophages. Cells were treated by polySia avDP20. PolySia avDP20 reduced the uptake of neural debris at a concentration of 1.5 μM. Data are presented as mean +/− SEM of n = 6 independent experiments. **p < 0.01; ANOVA followed by Bonferroni. (**c**) Release of superoxide as detected by DHE. Human macrophages both challenged with neural debris and pre-treated with different concentrations of polySia avDP20. Superoxide release triggered by neural debris was inhibited by 1.5 μM of polySia avDP20. Data are presented as mean +/− SEM of n = 3 independent experiments. **p < 0.01; ANOVA followed by Bonferroni. (**d**) Scavenging of the superoxide release by superoxide dismutase SOD1 and Trolox. Human macrophages were challenged with debris and superoxide release was determined by DHE. The release of superoxide was scavenged by addition of SOD1 or Trolox. The radical scavenger Trolox and the SOD1 confirmed that DHE detected extracellular production of superoxide. Data are presented as mean +/− SEM of n = 4 independent experiments. ***p < 0.001; ANOVA followed by Bonferroni.

Siglecs have been shown to block the oxidative burst associated with phagocytosis of neural debris in a murine culture system^12^ and *in vivo* in Siglec-E deficient mice^13^. To study the effect of polySia avDP20 on phagocytosis of neural debris and the associated oxidative burst, we incubated human macrophages with fluorescently labeled neural debris. First, we determined the uptake of neural debris into the cells by confocal microscopy and 3D-reconstruction. We observed ingestion of neural debris into macrophages (Fig. 5a). After treatment with polySia avDP20 the number of macrophages showing uptake of neural debris was slightly decreased (Fig. 5b). In detail, 1.5 μM polySia avDP20 reduced the relative uptake of neural debris by macrophages from 1 +/− 0.06 to 0.7 +/− 0.07 (p = 0.007; Fig. 5b).

To determine the phagocytosis associated oxidative burst, we used the superoxide-sensitive fluorescent dye dihydroethidium (DHE). Treatment of macrophages with neural debris stimulated the relative superoxide production from 1 +/− 0.09 to 1.6 +/− 0.1 (p = 0.004), while 1.5 μM polySia avDP20 completely prevented the debris induced stimulation of superoxide (0.9 +/− 0.09; p = 0.003; Fig. 5c). As a control we scavenged the superoxide by addition of superoxide dismutase-1 (SOD1) or by addition of Trolox, a water-soluble analog of vitamin E, into the medium. Both scavengers neutralized the superoxide that was induced by neural debris (Fig. 5d). The inhibitory effect of 1.5 μM polySia avDP20 on superoxide was as strong as the scavengers Trolox or SOD1 (Fig. 5d).

**Figure 6:**
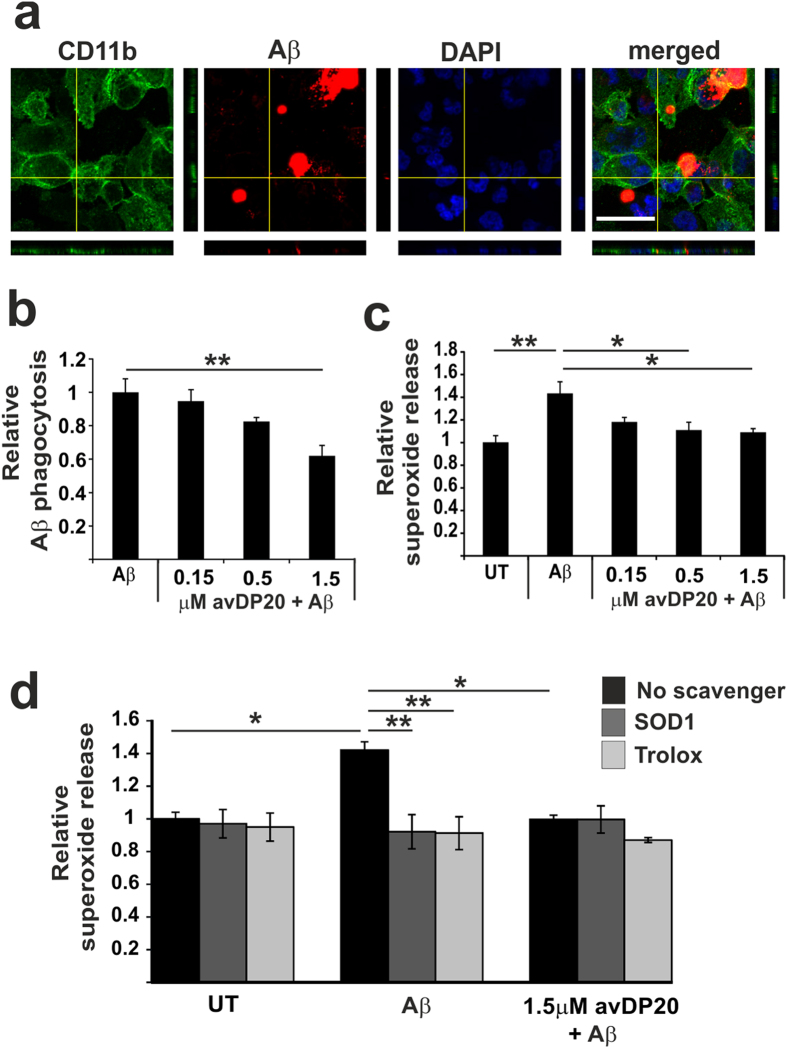
PolySia reduced macrophages phagocytosis of fibrillary amyloid-β_1–42_ and prevented the associated oxidative burst. (**a**) Phagocytosis of fibrillary biotin-conjugated amyloid-β_1–42_ by human macrophages. Cells were fixed and stained with fluorescent labelled streptavidin and an antibody directed against CD11b. Nuclei were labelled by DAPI. Confocal 3D-reconstruction of a macrophage cell that showed ingested amyloid-β_1–42_ material inside the cell. Scale bar: 50 μm. (**b**) Quantification of the uptake of amyloid-β_1–42_ into macrophages. Cells were treated for 1 hour by polySia avDP20. PolySia avDP20 prevented the uptake of amyloid-β_1–42_ at a concentration of 1.5 μM. Data are presented as mean +/− SEM of n = 5 independent experiments. **p < 0.01, ANOVA followed by Bonferroni. (**c**) Release of superoxide as detected by DHE of the human macrophages challenged with fibrillary amyloid-β_1–42_ and treated with different concentrations of polySia avDP20. Superoxide release triggered by amyloid-β_1–42_ was inhibited by 0.5 μM and 1.5 μM polySia avDP20. Data are presented as mean +/− SEM of n = 6 independent experiments. * < 0.05, **p < 0.01, ANOVA followed by Bonferroni. (**d**) Scavenging of the superoxide release by superoxide dismutase SOD1 and Trolox. Human macrophages were challenged with fibrillary amyloid-β_1–42_ and superoxide release was determined by DHE. The radical scavenger Trolox and the SOD1 confirmed that DHE detected extracellular production of superoxide. Data are presented as mean +/− SEM of n = 3 independent experiments. * < 0.05, **p < 0.01; ANOVA followed by Bonferroni.

We also determined the effect of polySia avDP20 on the uptake and superoxide production triggered by the Alzheimer´s disease associated fibrillary amyloid-β_1–42_. We incubated human macrophages with biotinylated fibrillary amyloid-β_1–42_ and visualized the uptake into the cells by confocal microscopy and 3D-reconstruction (Fig. 6a). PolySia avDP20 slightly reduced the uptake of amyloid-β_1–42_ into macrophages. In detail, 1.5 μM polySia avDP20 reduced relative uptake of fibrillary amyloid-β_1–42_ from 1 +/− 0.08 to 0.61 +/− 0.06 (p = 0.003; Fig. 6b). Furthermore, we analyzed the oxidative burst triggered by amyloid-β_1–42_. Again, production of superoxide release was determined via the fluorescence dye DHE. Treatment of macrophages with amyloid-β_1–42_ stimulated the relative superoxide production from 1 +/− 0.05 to 1.4 +/− 0.1 (p = 0.004), while 1.5 μM polySia avDP20 prevented the amyloid-β_1–42_ induced stimulation of the superoxide production (1.08 +/− 0.03; p = 0.03; Fig. 6c). SOD1 and Trolox scavenged the amyloid-β_1–42_ triggered superoxide molecules (Fig. 6d). Again, the inhibitory effect of polySia avDP20 on fibrillary amyloid-β_1–42_ was as strong as the scavengers.

In summary, polySia avDP20 reduced the phagocytosis of neural debris and fibrillary amyloid-β_1–42_ and completely inhibited the associated superoxide release.

### Neuroprotective activity of low molecular weight polySia

**Figure 7:**
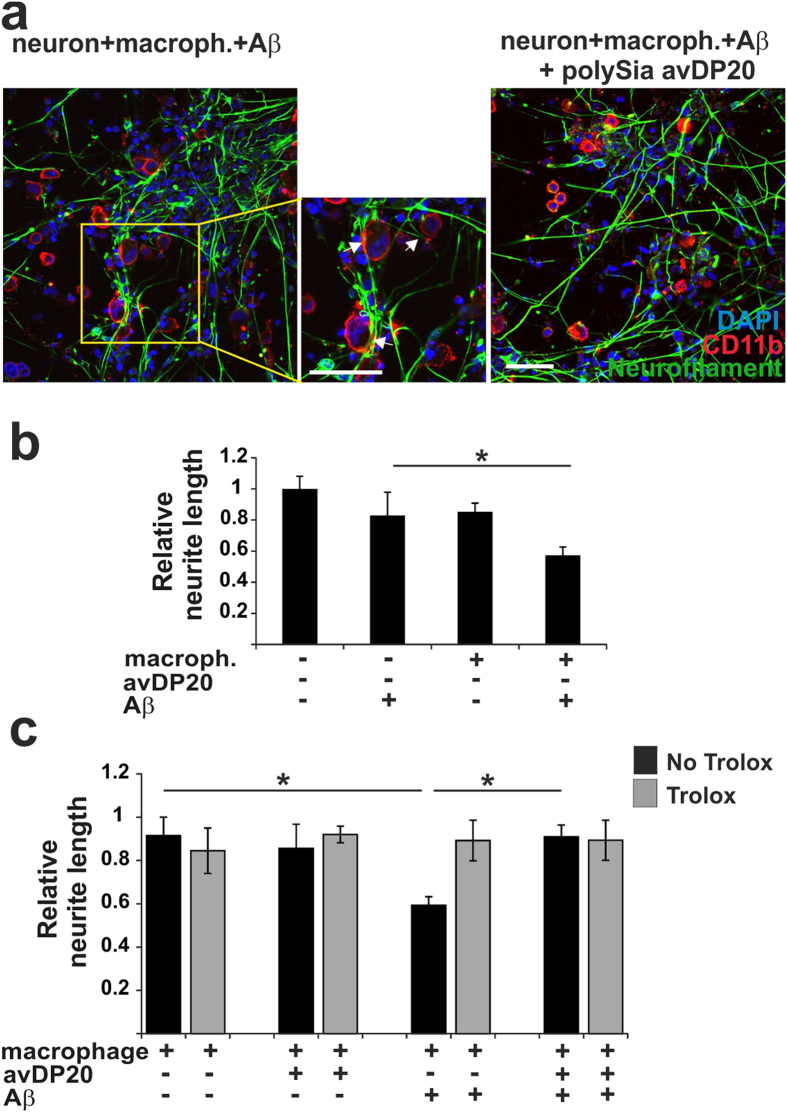
Neuroprotective effect of polySia with average degree of polymerization 20. (**a**) Co-culture of human macrophages and neurons. Cells were double-immunostained with antibodies directed against the macrophage marker CD11b and the neuronal marker neurofilament. Nuclei were labelled by DAPI. Co-cultures were treated with fibrillary amyloid-β_1–42_ and polySia avDP20 plus fibrillary amyloid-β_1–42_. Loss of neurites observed in amyloid-β_1–42_ treated co-cultures was prevented by addition of polySia avDP20. Scale bar: 50 μm. (**b**) Relative neurite length of human neurons was determined with or without addition of THP1 macrophages and/or fibrillary amyloid-β_1–42_ after 48 hours. Addition of amyloid-β_1–42_ alone did not affect the relative neurite length. Treatment of macrophage-neuron co-culture with amyloid-β_1–42_ further reduced the relative neurite length. Data are presented as mean +/− SEM of n = 3 independent experiments. *p < 0.05; ANOVA followed by Bonferroni. (**c**) Relative neurite length of human neurons in macrophage-neuron co-culture. The co-culture was incubated with fibrillary amyloid-β_1–42_ and/or polySia avDP20. The amyloid-β_1–42_ induced reduction in the relative neurite length was antagonized by polySia avDP20. Data are presented as mean +/− SEM of n = 3 independent experiments. *p < 0.05; ANOVA followed by Bonferroni.

Next, we tested polySia avDP20 in a co-culture of human macrophage and neurons. THP1 derived macrophage cells were added to the neurons and neurite length was determined after 48 hours of co-culture (Fig. 7a). Addition of macrophages to neurons slightly decreased the relative neurite length (Fig. 7b). Addition of fibrillary amyloid-β_1–42_ to macrophage-neuron co-culture further reduced the relative neurite length (Fig. 7b). In detail, relative neurite length was reduced from 1 +/− 0.08 to 0.59 +/− 0.03 after addition of macrophages and amyloid-β_1–42_. At the applied concentration of 1 μM amyloid-β_1–42_ alone had no neurite reducing effect (Fig. 7b). Next, we added polySia avDP20 (1.5 μM) to the macrophage-neuron co-culture. PolySia avDP20 did not interfere with the general neurite-reducing effect of the macrophages, but it completely inhibited the neurite-reducing effect of fibrillary amyloid-β_1–42_ (Fig. 7c). In detail, addition of fibrillary amyloid-β_1–42_ reduced the relative neurite length from 0.91 +/− 0.08 to 0.59 +/− 0.03 (p = 0.034), while treatment with polySia avDP20 antagonized this neurotoxic effect (0.9 +/− 0.05; p = 0.036; Fig. 7c). Trolox was added to the co-culture as a control to scavenge the radical-mediated neurotoxic effect. Trolox completely antagonized all neurite reducing effects of macrophages.

Thus, polySia avDP20 prevented the negative effects mediated by fibrillary amyloid-β_1–42_ and radicals on the neurite length of human neurons.

## Discussion

Here we show that low molecular weight polySia (avDP20) acted anti-inflammatory on human THP1 macrophages by inhibiting the LPS-induced gene transcription and protein secretion of TNFSF2 and by preventing the oxidative burst associated with phagocytosis of debris or fibrillary amyloid-β_1–42_. Furthermore, we demonstrate that the anti-inflammatory effect of polySia avDP20 on human macrophages is mediated via SIGLEC-11 receptors. PolySia avDP20 interacting with SIGLEC-11 as determined by ELISA and lentiviral knock-down of *SIGLEC11* neutralized the anti-inflammatory effect of polySia avDP20 on *TNFSF2* gene transcription and protein secretion. Thus, it is likely that the anti-inflammatory effect of polySia avDP20 is mediated via SIGLEC-11, although we cannot exclude that polySia also might act on other SIGLEC receptors of monocytes/macrophages recognizing α2.8-linked Sia (e.g. SIGLEC-5 or SIGLEC-7). SIGLEC-11 has a immunoreceptor tyrosine-based inhibitory motif (ITIM) in the cytosolic tail^2^, which can counteract the immunoreceptor tyrosine-based activation motif (ITAM) signalling of DAP12/TYROBP^14^. Upon target recognition by TYROBP-associated phagocytic receptors, the tyrosine of the ITAM will become phosphorylated and activates a series of downstream events, which trigger phagocytosis and NADPH-oxidase mediated release of superoxide^14^,^15^. This activatory signaling is counter-regulated by the ITIM containing SIGLEC receptors, which upon ligand binding could inhibit downstream signaling by de-phosphorylation of ITAM tyrosine residues^11^,^14^.

Expression of SIGLEC-11 was detected in human tissue on specialized tissue macrophages including microglia of the brain, Kupffer cells of the liver, lamina propria macrophages of the intestine and perifollicular cells in the spleen^2^. Furthermore, expression of SIGLEC-11 was immunostained in macrophages invading inflammatory tissue of the stomach, the tonsil or appendix^2^. However, it is still unclear how SIGLEC-11 is regulated in human macrophages and how far the THP1-derived macrophage model used in this study can reflect a defined polarization status of macrophages. In cultured murine microglial cells, that ectopically were expressing human SIGLEC-11, the LPS induced gene transcription of the pro-inflammatory mediators, interleukin-1β (IL-1β) and nitric oxide synthase-2 (NOS2) were reduced after artificial stimulation of SIGLEC-11 with cross-linking antibodies^3^. Furthermore, SIGLEC-11 expression on murine microglia had a neuroprotective effect, which was diminished by sialidase (EndoN) treatment of neurons that led to the removal of α2.8-linked Sias from the cell surface^3^. Thus, our current data with an anti-inflammatory action of polySia avDP20 on human SIGLEC-11 expressing macrophages are in line with these recent *in vitro* findings.

The effect of polySia avDP20 could be explained by multimerization of several SIGLEC-11 receptors. Normally, interaction between a single protein such as a SIGLEC receptor and a carbohydrate such as Sia is of relative low affinity, often insufficient for induction of a biological signal^16^,^17^. Therefore, it is necessary that several protein receptors pair together in a way that the carbohydrate ligands are recognized by a cluster of receptors^14^. This multivalency seems to be essential for protein-carbohydrate interaction and response in a cellular system^16^,^17^. Thus, two or more multimerized SIGLEC-11 receptors might recognize one stretch of polySia that is then leading to a biological effect. Short oligoSia would fail to induce such a multimerization of SIGLEC-11 receptors. Indeed, our data show that oligoSia DP6 did not antagonize the LPS-induced increase of *TNFSF2* gene transcripts in the human macrophages.

PolySia avDP20 also modulated the inflammation associated endocytosis/ phagocytosis. Microbeads are normally ingested by phagocytic receptors, while nanobeads are mainly taken up by receptor-mediated endocytosis. Interestingly, polySia avDP20 antagonized the LPS-triggered increase in phagocytosis without affecting the basal homeostatic phagocytosis and receptor-mediated endocytosis. While beads allow a distinction between basal and inflammation-triggered phagocytosis, debris and fibrillary amyloid-β_1–42_ always trigger an inflammatory response in macrophages. Thus, the slightly reduced uptake of debris and fibrillary amyloid-β_1–42_ by treatment with polySia avDP20 could be explained by antagonization of the inflammation-triggered phagocytosis. The finding that polySia avDP20 binding to SIGLEC-11 interferes with inflammatory phagocytosis is in agreement with our recent analysis on cultured mouse microglia, where we showed that ectopic expression of SIGLEC-11 in mouse microglia slightly reduced the uptake of debris^3^. Phagocytosis can be a defense mechanism to remove invading pathogens. In contrast, receptor-mediated endocytosis enables homeostatic uptake of macromolecules into the cells that will be re-utilized. Thus, the discrete effects of polySia avDP20 on inflammatory phagocytosis and receptor-mediated endocytosis as well as basal phagocytosis suggest a role of SIGLEC-11 in recognition of endogenous structures and anti-inflammatory signaling.

PolySia avDP20 completely prevented the superoxide release triggered by neural debris or fibrillary amyloid-β_1–42_. Recently, mouse Siglec-E has been shown to inhibit the production of superoxide radicals of phagocytes *in vitro* and *in vivo*^12^,^13^. Thus, human SIGLEC-11 acts similar to mouse Siglec-E suggesting that the ITIM-signaling receptors negatively interfere with the oxidative burst. While polySia avDP20 completely prevented the oxidative burst, phagocytosis was only partially reduced suggesting that polySia avDP20 still allows clearance of endogenous material without oxidative burst.

PolySia avDP20 acted neuroprotective in a fibrillary amyloid-β_1–42_ challenged human macrophage-neuron co-culture system. Amyloid-β_1–42_ increased the neurite diminishing activity of human macrophages. This is in line with the literature, where it was shown that even relative low concentrations of amyloid-β can reduce neuronal numbers in the presence of microglia^18^. The neurotoxic effect of fibrillary amyloid-β_1–42_ was neutralized in our human co-culture system by polySia avDP20. Experiments with the radical scavenger Trolox indicate that the neurite diminishing effect of fibrillary amyloid-β_1–42_ triggered by macrophages was mediated by reactive oxygen species (ROS). Thus, the protective effect of polySia avDP20 in this co-culture system could be explained by prevention of the macrophages oxidative burst.

Our data indicate that polySia avDP20 might be used as a novel therapy for neuroinflammatory diseases by preventing the phagocytes superoxide production and still allowing basal phagocytosis and endocytosis of endogenous structures. PolySia has been used before in therapy approaches, mainly by improving the pharmacokinetics of recombinant therapeutic proteins and antibodies^19^,^20^,^21^. Therefore, therapeutic proteins were decorated with polySia by posttranslational modification with terminal Sias by enzymatic or chemical means^19^,^20^,^21^. The polysialylated conjugates had greater stability, lower immunogenicity, and an increased blood circulation time due to the chemico-physical properties of these hydrophilic long chain molecules.

Our data now demonstrate that low molecular weight polySia (avDP20) can interact with SIGLEC-11 and can act anti-inflammatory on SIGLEC-11 expressing human macrophages. Thus, polySia avDP20 is a novel interesting candidate for inflammatory diseases involving increased production of superoxide by macrophages.

## Methods

### Fragmentation and separation of polySia

Purified α2.8-linked polysialic acid (~50–70 kDa^8^,^9^; University of Hanover and Carbosynth Limited, UK) was used for fragmentation and anion exchange high-performance liquid chromatography (HPLC) separation and purification. Then strong anion exchange high-performance liquid chromatography (SAX-HPLC) was applied for its separation and purification. First, samples were heated at 65 °C for 90 minutes to induced spontaneous hydrolysis. Then, fragmented polySia was subjected to a 53 ml strong anion-exchange column (Hi Load 26/10 Q, GE-Healthcare) and separated via a HPLC system coupled to a photometric UV detector at 205/280 nm (Amersham Bioscience/GE Healthcare), utilizing a 2 M NH_4_HCO_3_ buffer as solvent with a flow-rate of 4 ml/minute (Table 1). The flow-through was collected in 90 tubes with a respective volume of 8 ml. Each three consecutive tubes were pooled to get 30 fractions. Fractions 3–4 were collected and analyzed by gel electrophoresis. PolySia with average degree of polymerization (here labeled as polySia avDP20) was selected for further experiments. To get rid of buffer residues the samples were lyophilized and solved in water (Ampuwa, Fresenius).

### Determination of the polySia concentration

Determination of the concentration of polySia avDP20 was performed with a thiobarbituric acid based method^22^. Therefore, polySia was pretreated with 1 M H_2_SO_4_ at 80 °C for 1 hour in order to hydrolyze the polymer into single n-acetylneuraminic acid (Sia monomers). A standard containing concentrations form 0–50 μg Sia monomers (Nacalai Tesque ING, Japan) was prepared. The test samples and the standard were treated with 25 μl of 25 mM periodic acid in 0.125 M H_2_SO_4_ and incubated at 37 °C for 30 minutes. Afterwards, 20 μl of 2% sodium arsenite solution (in 0.5 N HCl) was added to each sample to reduce the excess of periodate. After 2 minutes at room temperature, 200 μl of 2-thiobarbituric acid (0.1 M, pH 9) was given to the samples. Subsequently, a heating step (7.5 minutes at 99 °C) was performed to induce the formation of a red colored complex. The solution was cooled on ice for 5 minutes, afterwards shaken with 500 μl/sample acid butanol (butan-1-ol plus 5% of 12 N HCl) and then centrifuged to separate the two phases. The intensity of the colorful upper phase was measured via spectrometer at 549 nm. Quantification was performed based on the Sia monomers standard.

### Gel electrophoresis of polySia

PolySia avDP20 was analyzed by 18% Tris-Glycine polyacrylamide gel (Life Technologies GmbH; 2.5 hours electrophoresis at 125 V). Sulphated dextrans of different sizes were used as marker (TdB Consultancy). Subsequently, the gel was stained via a protocol from Goldberg and Warner for at least 2 hours at room temperature with Stains-All (Sigma) solution (30 mM Tris, 25% isopropanol, 7.5% formamide and 0.025% (w/v) at a pH of 8.8). To clear the background, the gel was washed with deionized water including 25% isopropanol afterwards.

### Analytic HPLC analysis of oligoSia DP6 and polySia with avDP20

Oligosialic acid with a degree of polymerization of 6 (oligoSia DP6) was obtained from Nacalai Tesque, Japan. The labeling of oligoSia DP6, polySia avDP18, avDP20, avDP22 and hydrolyzed colominic acid with 1,2-diamino-4,5-methylenedioxybenzenewas (DMB) was carried out over at least 48 hours at 4 °C as previously described by Inoue *et al.*^23^. Before the run the sample was set to a pH of 8 to improve binding of the Sia to the column. HPLC retention time was monitored via a Mono Q 5/50 column operated with a flow speed of 0.5 ml/minute (GE Healthcare) in a linear gradient from 0–300 mM NaCl in order to elute and separate the single fractions of polySia avDP18, avDP20 and avDP22.

### Determination of the interaction between SIGLEC-11 and polySia avDP20 by ELISA

The polySia avDP20 was coupled with a biotin molecule at the terminus of the polySia avDP20 chain. Therefore, polySia with avDP20 was oxidized to an aldehyde by sodium metaperiodate. Afterwards, the hydrazide coupled biotin (Thermo Scientific) was bound at room temperature to the aldehyde group to form a hydrazone bond. Purification was carried out with a desalting column (HiTrap Desalting Column GE Healthcare). A 96-well plate was coated with 10 μg/ml pierce recombinant protein-A (Thermo Scientific) and incubated overnight at 4 °C. All following steps were carried out at room temperature. The plate was washed 3 times with PBS+ 0.05% tween20 and blocked with 3% BSA for 1 hour. Blocking solution was removed and 5 μg/ml of recombinant human SIGLEC-11/Fc chimera (R&D system) was added to the wells and incubated for 2 hours. Next, the plate was washed 3 times with PBS+ 0.05% tween20 and blocked with 3% BSA for 1 hour. Afterwards, blocking solution was removed and different concentrations of biotinylated-polySia avDP20 (0.01, 0.05, 0.25, 1.25, 6.25 μg/ml) or biotinylated dextran (5 kDa, NANOCS) serving as control (0.01, 0.05, 0.25, 1.25, 6.25 μg/ml diluted in PBS) were added and incubated for 2 hours. The plate was washed 3 times with PBS + 0.05% tween20 and incubated with streptavidin-HRP (0.5 μl/ml, BD Bioscences, Pharmingen) for 1 hour, followed by 3 times washing with PBS+ 0.05% tween20. At the end 100 μl TMB (Sigma) were added for 15 minutes and the reaction was stopped by 100 μl 1N HCL. The signal was measured at 450 nm by an ELISA plate reader (PerkinElmer).

### Flow cytometry analysis

A novel SIGLEC-11 specific mouse monoclonal antibody (clone 3 EH) binding up to a dilution of 1:128 K to the SIGLEC-11 specific peptide ISISHDNTSALE was generated by Abmart. To confirm the binding specificity of the antibody, HEK293-T cells were transfected with plenti-EF1α-*SIGLEC11-* or -*SIGLEC16*-IRES-GFP over-expression plasmid as well as a plenti-EF1α-IRES-GFP control plasmid. Transfection procedure was carried out using Lipofectamine 2000 (Invitrogen). In brief, 2.5 × 10^6^ cells were plated on 10 cm cell culture dishes. Regular cell culture medium was changed directly before the procedure to Opti-MEM medium with 10% FCS. The plasmids (5 μg) and Lipofectamine 2000 (90 μl) were combined according to the manufacturer’s instructions and added for 24 hours to the cells. Afterwards, cells were detached mechanically and washed with PBS. Cells were stained for protein expression by the SIGLEC-11 specific antibody (1:500) followed by Cy-5 fluorescence-labelled secondary antibody (Jackson Laboratories Inc, USA, 1:200). Control samples were incubated with an isotype control and secondary fluorescence labelled antibodies. Analysis was performed with a flow cytometer (BD, Calibur) and the FlowJo 8.7 Software (Tree Star Inc.).

### THP1 cell culture

The human monocytic cell line THP1 (kindly provided by V. Hornung, University of Bonn) derived from an acute monocytic leukemia patient was used to obtain macrophages. THP1 monocytes were cultured in medium containing RPMI (Gibco) supplemented with 10% defined fetal bovine serum (Gibco), 1% Penicillin-Streptomycin (Gibco), 1% L-glutamine (Gibco) and 1% sodium pyruvate (Gibco). Macrophage cultures were differentiated repeatedly from frozen stocks of THP1 cells. At least one week before experiment, THP1 monocytes were moved to THP1 differentiation medium containing RPMI with 1% Penicillin-Streptomycin, 1% L-glutamine, 1% sodium pyruvate plus 1% N2 supplement (Gibco) and 1% chicken serum (Gibco). To differentiate THP1 cells into a macrophage phenotype, the cells were treated in THP1 differentiation medium with 0.5 μg/ml phorbol-12-myristate-13-acetate (PMA, Sigma) for 3 hours. Afterwards, cells were washed 2 times with 37 °C warm medium and were kept in PMA-free THP1 differentiation medium for at least 24 hours. Then a serum free medium (THP1 differentiation medium without chicken serum) was applied to the macrophages.

### Phagocytosis of microbeads and endocytosis of nanobeads

To investigate phagocytosis activity, macrophages were plated/differentiated on 6-well-plates in a concentration of 2.5 × 10^5^ cells per well. Cells were stimulated for 24 hours with 1 μg/ml LPS (InvivoGen, *E.coli* 0111:B4 strain) prior to the addition of micro- or nanobeads. PolySia was added to the micro- or nanobeads and incubated at 4 °C. After 1 hour, beads (microbeads from Polyscience Inc., PE labeled, 1 μm bead diameter; nanobeads from Life technologies, yellow-green labeled, 0.04 μm bead diameter) were added in a concentration of 1 μl beads/ml medium (microbeads) or 0.1 μl beads/ml medium (nanobeads) for 1 hour (microbeads) or 5 minutes (nanobeads) to the cells. All steps were performed under regular cell culture conditions (37 °C, 5% CO_2_). For analysis, media was removed and cells were treated for 1–2 minutes with trypsin (Gibco) in order to get rid of beads sticking to the surface of macrophage cells. Afterwards cells were washed 3-times with PBS before being detached mechanically. In addition, nanobeads treated cells were fixed for 15 minutes with 2% paraformaldehyde (PFA) solution before flow cytometry analysis to stop endocytosis. Flow cytometry analysis was performed with a FACS Calibur (BD Biosciences).

### Determination of cell viability by the MTT-assay

Cell viability was determined by the 3-(4,5-Dimethylthiazol-2-yl)-2,5-diphenyltetrazolium bromide (MTT) assay (Millipore). Cells were plated on a 96 well plate (2.5 × 10^4^) treated for 24 hours with different concentrations of polySia avDP20. To generate physiologic conditions a pH of 7 was adjusted for polySia avDP20 at higher concentrations (15 μM or higher) using sodium hydroxide and 50 μM Tris. Additionally, the colour of phenol red in the culture medium was closely monitored to confirm absence of major pH changes. After 20 hours of stimulation, 10 μl of MTT-reagent was added to each well and cells were cultured for another 4 hours. Then, cells were lysed with isopropanol containing 0.04 N HCL to measure the dye formazan. The light absorbance of the purple formazan dye was determined by a spectrophotometer at a wavelength of 570 nm with a reference wavelength of 630 nm (Perkin Elmer, Envision Multiplate Reader). Macrophage cell numbers were determined via a Neubauer cell chamber. The data from the MTT-assay of the macrophages were normalized to the cell number. Measured absorbance values were divided through the number of cells per well to determine the turnover per single cell. All values were compared to unstimulated control cells to receive the relative changes of cell proliferation and viability.

### Gene transcript analysis of *SIGLEC7*, *SIGLEC10, SIGLEC11* and *TNFSF2*

Total RNA was collected from cells via the RNeasy kit system (Quiagen). Reverse transcription of the RNA was performed using Super Script III reverse transcriptase (Life Technologies) and hexamer random primers (Roche). *SIGLEC7* (for 5′-GCTCAGCCTCTCAGGGTAAC-3′, rev 5′-GGGTCGTGAACCCTCAAACA -3′)*, SIGLEC10* (for 5′-GGACTCAGACAGAAACCCCG-3′, rev 5′-TGTGGCTTTCTGATTCCGCT-3′) and *SIGLEC11* (for 5′-CACTGGAAGCTGGAGCATGG-3′, rev 5′-ATTCATGCTGGTGACCCTGG-3′) PCR was performed at Tm of 60 °C for 35 cycles with *GAPDH* as loading control (for 5′-CTGCACCACCAACTGCTTAG-3′, rev 5′-TTCAGCTCAGGGATGACCTT-3′). Quantitative qRT-PCR with specific oligonucleotides was performed with SYBR Green PCR Master Mix (Qiagen) using the ABI 5700 Sequence Detection System (PerkinElmer). The qRT-PCR was running for 40 cycles with a Tm of 60 °C using the same primer as for the PCR plus *TNFSF2* (for 5′-GACAAG CCTGTAGCCCATGT-3′ rev 5′-AGGAC CTGGGAGTAGATGAGG-3′). The δδCT method with *GAPDH* as internal standard was performed for qRT- PCR quantification.

### Plasmid construction, viral particle production and lentiviral transduction

Plasmids for lentiviral knock-down of *SIGLEC11* (shRNASig11: TRCN0000062842, Open Biosystems) were obtained from a knock-down library in a human pLKO.1 lentiviral shRNA target gene set backbone (Open Biosystems). A pLenti 6.2/V5_DEST Gateway Vector (Life technologies) without or scrambled target gene served as control vector. For lentiviral virus production, HEK293-FT cells (Invitrogen) were transfected with the targeting and packaging plasmids (Addgene). For transfection, calcium chloride (CaCl_2_) was used. Medium was changed 16 hours post-transfection. Supernatant was collected 48 hours after transfection. For concentration, supernatant containing the viral particles was incubated for 1.5 hours with 50% Polyethylene glycol (Roth GmbH), 4 M NaCl and 1xPBS at 4 °C. Subsequently the solution was centrifuged with 4500 g at 4 °C for at least 30 minutes. The viral particle containing pellet was resuspended in 1 ml of medium and added to the target cells. After 48 hours of incubation transduced cells were selected by adding 1 μg/ml puromycin. The knock-down efficiency was determined by qRT-PCR and flow cytometry.

### TNFSF2 protein detection by ELISA

For the detection of TNFSF2 release by THP1 cells a Quantikine ELISA kit (R&D Systems) was used. Macrophages were stimulated with 1 or 50 μg/ml LPS and 1.5 μM polySia avDP20 for 24 hours. Supernatant was harvested and used according to the manufacturer’s instructions. Optical density of the ELISA samples was determined by a spectrophotometer at a wavelength of 450 nm with a reference wavelength of 560 nm (Perkin Elmer, Envision Multiplate Reader).

### Generation of human neurons from iPS cell lines

Human iPS cell line (Foreskin-1, WiCell^24^) was used for generation of neurons out of primitive neural stem cells (pNSCs). Therefore, iPS cells were cultured on feeder cells to form small colonies. Next, medium was changed to neural stem cell medium (DMEM/F12: Neurobasal; GIBCO) in the presence of leukaemia inhibiting factor (LIF; Millipore, 10 ng/ml) and three small molecules CHIR99021 (inhibitor of GSK-3β, Axon Medchem, 4 μM) and SB431542 (inhibitor of TGF-β and activin receptors; Axon Medchem, 3 μM), and Compound E (inhibitor of γ-secretase; Axon Medchem, 0.1 μM) for 10 days. To induce differentiation towards neurons, pNSCs were dissociated by accutase (PAA) and cultured on poly-L-ornithine (Sigma, 0.15 mg/ml) plus laminin (Sigma, 1 μg/ml) coated cell culture dishes in neural stem cell medium (DMEM/F12:Neurobasal, plus LIF, CHIR99021 and SB431542) till cell attached and formed small colonies. Then, medium was changed to neuronal differentiation medium (DMEM/F12, plus N2 and B27 supplements, GIBCO) plus ascorbic acid (TOCRIS, 0.2 mM) and cyclic adenosine monophosphate (Sigma, 300 ng/ml) in presence of brain derived neurotrophic factor (BDNF; Prospect, 10 ng/ml) and glial cell line-derived neurotrophic factor (GDNF; Prospect, 10 ng/ml) for 2 weeks. Medium containing the neurotrophic factors was changed every second day.

### Phagocytosis of debris

pNSCs from an established induced pluripotent (iPS) stem cell line (WiCell, foreskin-1)^24^ as described above were incubated with 40 nM okadaic acid (Sigma) for 24 hours. Then, cellular debris was collected and centrifuged. Debris was washed with PBS and stained with “Dil-Derivatives for Long-Term Cellular Labeling” Molecular Probes (Invitrogen) according to the supplier´s manual. Macrophages were pre-incubated for 1 hour with different concentrations of polySia avDP20 (0.15, 0.5 and 1.5 μM) followed by 1.5 hour incubation with 5 μg/ μl pre-stained cell debris. Cells were fixed for 15 minutes with 4%PFA, blocked and permeabilized for 60 minutes with a solution that contained bovine serum albumin (10% BSA) and normal goat serum (5% nGS) and 0.1% TritonX-100. Then cells were stained with a CD11b antibody (Becton Dickinson) followed by a secondary Alexa488-conjugated antibody (Molecular probes). For analysis, images were randomly scanned and 3D-reconstructions were obtained by a confocal laser scanning microscope (Fluoview 1000, Olympus). To determine the ratio of cells having ingested fluorescently labeled material, five images per experiment group were obtained and all cells on the images were quantified using Image J software (NIH).

### Phagocytosis of fibrillary amyloid-β_1–42_

To obtain fibrillary amyloid-β_1–42_ forms, biotinylated amyloid-β_1–42_ (Bachem) was incubated for 72 hours at 37 °C as previously described^25^. Macrophages were pre-incubated for 1 hour with different concentrations of polySia avDP20 (0.15, 0.5 and 1.5 μM), followed by 1.5 hour incubation with 2 μM fibrillary biotinylated amyloid-β_1–42_. Cells were fixed for 15 minutes with 4% PFA, blocked and permeabilized for 60 minutes with a solution that contained 10% BSA and 5% nGS and 0.1% TritonX-100. Afterwards, cells were incubated with a CD11b antibody (Becton Dickinson) followed by a secondary Alexa 488-conjugated antibody directed against rat IgG (Molecular probes) and streptavidin-Cy3 (Jackson ImmunoResearch). For analysis, images were randomly obtained with a confocal laser scanning microscope and the fluorescent labeled amyloid-β_1–42_ was visualized inside macrophages by 3D-reconstruction (Fluoview 1000, Olympus). To determine the ratio of cells having ingested fluorescent marked amyloid-β_1–42_, five images per experimental group were collected and all cells of the images were analyzed using Image J software (NIH).

### Detection of superoxide by dihydroethidium

To measure the relative production of superoxide by macrophages, cells were differentiated in 4-chamber culture dishes. After 48 hours cells were treated with either 10 μM fibrillary amyloid-β_1–42_ or 5 μg/μl debris for 15 minutes with or without 1 hour polySia avDP20 pre-incubation. To test the antioxidant effect of SOD1 or Trolox as positive controls, macrophage cells were pre-incubated 1 hour either with 60 U/ml SOD1 (Serva) or 40 μM Trolox (Cayman), then fibrillary amyloid-β_1–42_ or debris added to them. Afterwards, cells were washed 2-times with Krebs-HEPES-buffer and afterwards incubated for 15 minutes with 30 μM DHE solution (diluted in Krebs-HEPES-buffer). Finally cells were washed 2-times with Krebs-HEPES-buffer and fixed for 15 minutes with 0.25% glutaraldehyde and 4% PFA. In total, three images were randomly collected per experimental group by confocal laser scanning microscopy (Fluoview 1000, Olympus). All cells of the collected images were analyzed by Image J software (NIH).

### Macrophage-neuron co-culture and analysis by immunocytochemistry

THP1 macrophage cells (ratio of 1:5 of macrophage:neurons) and 1 μM fibrillary amyloid-β_1–42_ were added to the iPS cell-derived neurons (see above) with or without polySia avDP20 for 48 hours in differentiation medium without growth factors. To use Trolox as a positive control, 40 μM Trolox were added at the same time with or without amyloid-β or polySia avDP20. Cells were fixed for 15 minutes with 4% PFA, blocked and permeabilized for 60 minutes with a solution that contained 10% BSA and 5% nGS and 0.1% TritonX-100. Next, immunostained with monoclonal rat anti-CD11b (BD pharmingen) and neurofilament (Sigma) antibodies overnight at 4 °C followed by secondary Cy3-conjugated goat antibody directed against rat IgG (Dianova) Alexa488-conjugated antibody directed against rabbit IgG (Molecular Probes) for 2 hours at room temperature. five images were randomly collected from each experimental setup by confocal laser scanning microscopy (Fluoview 1000, Olympus) and total lengths of neuronal branches from neurofilament stained neurites was determined by the NIH ImageJ/NeuronJ software.

### Statistical analysis

Normal distribution of data was checked by Kolmogorov-Smirnov test. Levene’s test was used to check equal distribution of data. Data are presented as mean +/− SEM of at least 3 independent experiments. Data were analyzed by student t-test for experiments with two groups only and by one-way ANOVA followed by Bonferroni test (when equal variances were assumed) using SPSS20 software.

### Use of human cell lines

All experiments were performed in accordance with relevant guidelines and regulations. All experiments involving established and commercially available human cell lines have been performed in accordance with the ethical guidelines of the University of Bonn and the national legislations.

## Additional Information

**How to cite this article**: Shahraz, A. *et al.* Anti-inflammatory activity of low molecular weight polysialic acid on human macrophages. *Sci. Rep.* **5**, 16800; doi: 10.1038/srep16800 (2015).

## Figures and Tables

**Table 1:** Elution conditions of the HPLC for separation of the polySia fractions.

elution volume NH_4_HCO_3_		elution time NH_4_HCO_3_	
in ml	in %	in minutes	in %
0	30	0	30
110	50	440	50
115	100	460	100
125	100	500	100
130	0	520	0
160	0	640	0
